# Recognition of pivotal immune genes NR1H4 and IL4R as diagnostic biomarkers in distinguishing ovarian clear cell cancer from high-grade serous cancer

**DOI:** 10.3389/fmolb.2025.1600808

**Published:** 2025-06-27

**Authors:** Yumin Ke, Meili Liang, Zhimei Zhou, Yajing Xie, Li Huang, Liying Sheng, Yueli Wang, Xinyan Zhou, Zhuna Wu

**Affiliations:** ^1^ Department of Gynecology and Obstetrics, The Second Affiliated Hospital of Fujian Medical University, Quanzhou, Fujian, China; ^2^ Department of Gynecology and Obstetrics, Qingtongxia People’s Hospital, Wuzhong, China

**Keywords:** bioinformatics analysis, NR1H4, IL4R, immune infiltration, ovarian clear cell carcinoma

## Abstract

**Objective:**

Ovarian clear cell carcinoma (OCCC) is characterized by poor prognosis and limited early diagnostic markers. Identifying molecular distinctions between OCCC and the more common high-grade serous ovarian cancer (HGSC) is critical to developing targeted diagnostic and therapeutic strategies for improved clinical outcomes.

**Methods:**

We retrieved the mRNA expression profiles of OCCC and HGSC from the Gene Expression Omnibus (GEO) database. To identify differentially immune-related genes (DIRGs) linked to OCCC. We assessed DIRGs functional enrichment and built a protein-protein interaction (PPI) to explore DIRGs interactions. Least Absolute Shrinkage and Selection Operator (LASSO) regression model and Multiple Support Vector Machine Recursive Feature Elimination (mSVM-RFE) methods were applied to identify predictive genes. The diagnostic performance of these candidate genes was evaluated using receiver operating characteristic (ROC) curves. A nomogram was constructed to predict OCCC. We further validated key DIRGs’ diagnostic ability via a validation set and immunohistochemistry (IHC). The CIBERSORT algorithm was used to analyze correlations between DIRGs and immune cell types in OCCC.

**Results:**

We detected 10 DIRGs in OCCC compared to HGSC. These genes were mainly linked to collagen-rich extracellular matrix, Phosphoinositide-3 Kinase- Protein Kinase B (PI3K-AKT) pathway, and transcriptional dysregulation in cancer. Nuclear receptor subfamily 1 group H member 4 (NR1H4) and Interleukin-4 Receptor (IL4R) emerged as potential biomarkers for OCCC (AUC_NR1H4_ = 0.809; AUC_IL4R_ = 0.840). In the validation cohort, AUC_NR1H4_ = 0.848 and AUC_IL4R_ = 0.821, respectively. IHC revealed higher expression levels of NR1H4 and IL4R in OCCC (P < 0.05). Additionally, NR1H4 correlated positively with resting memory T cells and neutrophils, while IL4R correlated with resting Natural Killer (NK) cells and neutrophils.

**Conclusion:**

NR1H4 and IL4R are promising immune-related diagnostic biomarkers for OCCC, with potential roles in neutrophil-mediated tumor microenvironment modulation. These findings enhance understanding of OCCC pathogenesis and provide a foundation for developing targeted diagnostic tools and immunotherapeutic strategies.

## 1 Introduction

Ovarian epithelial malignancy is a prevalent neoplasm in women worldwide, ranking seventh among all malignant tumors (Siegel et al., 2020). It can be categorized into five main histological subtypes with distinct histopathological features and molecular pathogenesis: high-grade serous carcinoma, endometrioid carcinoma, clear cell carcinoma, mucinous carcinoma, and low-grade serous carcinoma. HGSC is the most frequently observed subtype, accounting for 60% of all cases of epithelial ovarian malignancy. OCCC is the second most common subtype, representing 5%–11% of all ovarian epithelial malignancies (Zhu et al., 2021; Torre et al., 2018). On Magnetic Resonance Imaging (MRI), OCCC and HGSC can present as large pelvic cystic masses with solid components, exhibiting regular or irregular shapes. The cystic lesions may appear as single or multiple entities, some of which are separated, and the cyst wall may exhibit masses, nodules, or papillae protruding into the cavity. The solid components mostly demonstrate moderate to marked enhancement during contrast-enhanced scanning. Abdominal fluid accumulation is commonly observed. However, in comparison to HGSC, OCCC typically presents with a more regular shape, larger diameter, unilateral onset, and a higher prevalence of International Federation of Gynecology and Obstetrics (FIGO) stage I disease. Most OCCC tumors exhibit characteristics of cystic-solid composition with predominantly single cysts and internal prominences that are mainly nodular or papillary. High signal intensity within the cystic component on T2-weighted imaging (TWI) is more frequently encountered in OCCC cases, whereas low signal intensity within the solid component on TWI is more common. Furthermore, peritoneal implantation metastases are rare in OCCC cases, while peritoneal effusion may occur.

In contrast to HGSC, OCCC typically lacks P53 mutations and exhibits a lower frequency of Breast Cancer Gene (BRCA)1/2 mutations (6.3%). However, it has a higher incidence of AT-rich interaction domain 1A (ARID1A) and Phosphatidylinositol-4,5-bisphosphate 3 kinase catalytic subunit alpha (PIK3CA) mutation (Zhou et al., 2020). Both OCCC and HGSC are primarily managed through surgical intervention followed by adjuvant chemotherapy, however, OCCC has a poorer prognosis due to its resistance to conventional platinum-based drugs (Itamochi et al., 2008). It has been observed that Hepatocyte Nuclear Factor-1β(HNF-1)βis preferentially activated in OCCC, leading o immunosuppression within the tumor microenvironment through activation of the Signal Transducer and Activator of Transcription 3 (STAT-3) signaling pathway and Nuclear Factor-κB (NF-κB) dependent pathway, resulting in the production of Interleukin-6 (IL-6) and Interleukin-8 (IL-8). Overexpression of NF-κB signaling can be detected both in serum and cancerous ascites from OCCC patients, inducing inhibitory co-stimulators, Programmed Death - Ligand 1 (PD-L1), and B7-H1 as part of the B7 family molecules (Oda et al., 2018). Furthermore, gene expression profiling studies on OCCC tumors have revealed overexpression of Effector memory CD8^+^ T cells, cytotoxic T lymphocyte-associated antigen 4 (CTLA-4), PD-1, T cell immunoglobulin and mucin domain 3 (Tim-3), and lymphocyte activation gene 3 (LAG3). Conversely, human leukocyte antigen (HLA) expression was found to be decreased. These findings collectively contribute to an immunosuppressive microenvironment (Oda et al., 2018). Fortunately, the distinct immune microenvironment of OCCC has facilitated the emergence of immunotherapy as a novel therapeutic approach in recent years.

To enhance the prognostic outcomes for patients with OCCC, it is imperative to investigate the cellular and molecular distinctions between OCCC and HGSC to develop more efficacious treatment strategies specifically tailored for OCCC.

## 2 Materials and methods

### 2.1 Acquisition and analysis of microarray datasets

Initially, we accessed the matrix files for GSE63885, GSE73614, GSE65986, and GSE68600 from the GEO database (https://www.ncbi.nlm.nih.gov/gds). The GSE63885 and GSE73614 were used as the training groups. The GSE65986 and GSE68600 were used as test groups (Table 1). Subsequently, the microarray data downloaded from GEO were processed by converting the probe matrix into a gene matrix based on the annotation file. Subsequently, between-array normalization was performed using the normalizeBetweenArrays function from the “limma” R package, followed by batch effect correction using the ComBat algorithm implemented in the “sva” package. The tool available within the R software environment, as referenced on the Bioconductor website (http://www.bioconductor.org/) (Ritchie et al., 2015), where genes with logFCfilter = 0.585 and a corrected p-value< 0.05 were categorized as DEGs. A total of 1,509 immune-related genes (IRGs) were sourced from the ImmPort database (https://www.immport.org/shared/). The overlap between the DEGs and the IRGs derived from both datasets was utilized for further analytical processes.

**TABLE 1:** GEO database date of the OCCC and HGSC mRNA expression profile.

Data set ID	Platform	OCCC	HGSC
Train group			
GSE63885	GPL570	9	48
GSE73614	GPL6480	35	3
Test group			
GSE65986	GPL570	25	16
GSE68600	GPL80	8	32

### 2.2 GO and KEGG pathway enrichment analyses

To further elucidate the biological functions and underlying mechanisms of the previously identified DIRGs, we employed the “org.Hs.eg.db,” “ClusterProfiler,” “enrichplot,” “DOSE,” and “ComplexHeatmap” package in R to perform GO and KEGG pathway enrichment analyses (Wu et al., 2021). “org.Hs.eg.db” database was used for GO analysis, this database is a genomic annotation package for *Homo sapiens* (humans), which is used for gene identifier conversion (such as converting gene symbols to Entrez IDs) and functional annotation (such as GO enrichment analysis). “org.Hs.eg.db,” and KEGG Database (Online API). Directly accesses KEGG’s online interface via the clusterProfiler::enrichKEGG function (organism = “hsa” for human) to retrieve pathway enrichment information. Then the visualization of the enrichment outcomes was accomplished through the “ggplot2” package in R. The p-value filter <0.05 was considered statistically significant.

### 2.3 Construction of PPI network

We used the Search Tool for the Retrieval of Interacting Genes/Proteins (STRING) database to analyze the PPI of DIRGs. Then, the Cell Hubba plug-in in Cytoscape software was employed for clustering the network genes, and a PPI interaction network consisting of 10 nodes was obtained (von Mering et al., 2005).

### 2.4 Construction of a DIRG’s prediction model for OCCC

The Spearman correlation analysis was performed by screening the DIRG of |log fold change (FC)| = 1 and adj.P.Val.Filter less than 0.05 to investigate the correlation between these genes. To identify the significant prognostic biomarkers in OCCC, we utilized LASSO as well as the mSVM-RFE algorithm (Zhou and Tuck, 2007). The Lasso algorithm, a regression analysis technique implemented through the “glmnet” package, effectively filters variables to prevent overfitting. The mSVM-RFE algorithm improves the prioritization of features by incorporating resampling strategies throughout iterative processes, and it leverages supervised learning techniques to pinpoint key features. This is achieved by discarding feature vectors that are constructed through the mechanism of support vector machines.

The candidate genes obtained from both algorithms were intersected to confirm the prognostic candidate genes of OCCC. The “proc” R software was utilized to generate an ROC curve, and the AUC value was computed to assess the accuracy and effectiveness of the candidate genes.

### 2.5 Construction and verification of the nomogram model for the diagnosis of OCCC

To forecast the incidence of OCCC, we developed a Nomogram model utilizing the “rms” package. The “points” represent the assigned scores for the corresponding factors mentioned below, while the “total points” indicate the cumulative score of all the aforementioned factors. Subsequently, calibration was performed.

### 2.6 The diagnostic value of key DIRGs was verified

To further validate the diagnostic performance of the candidate key DIRGs, we employed validation sets GSE65986 and GSE68600. ROC curves were constructed, and the corresponding Area Under Curve (AUC) values were calculated using the “pROC” package in R software. Thereafter, immunohistochemistry staining was utilized to confirm the expression levels of IL4R and NR1H4 in OCCC and HGSC.

### 2.7 Patients and tissue samples

A sum of 14 paraffin-embedded specimens of OCCC and 47 HGSC were gathered from the Second Affiliated Hospital of Fujian Medical University from 2021 to 2023. The study was approved by the hospital’s Research Ethics Committee.

### 2.8 Immunohistochemistry

IHC staining was conducted by previously established protocols (Wu et al., 2022). The primary antibodies were anti-IL4R and anti-NR1H4 (AFFINTY, USA). Negative staining was assigned a score of 0, light yellow staining received a score of 1, brownish-yellow staining was scored 2, and tan staining was given a score of 3. The ratio of positively stained cells was assessed as follows: if less than one-third of the cells were positive, the score was 1; if between one-third and two-thirds were positive, the score was 2; if more than two-thirds were positive, the score was 3. The final score for IL4R and NR1H4 expression was calculated by multiplying the staining intensity score by the proportion score. Based on this final score, tissue sections were classified into low-expression (score <6) and high-expression (score ≥6) groups. Histopathological diagnoses were independently confirmed by two board-certified pathologists specializing in obstetrics and gynecology to ensure diagnostic accuracy and consistency.

### 2.9 Evaluation of infiltrating immune level

To analyze the immune cell composition in patients with OCCC, the CIBERSORT algorithm was utilized, leveraging gene expression data. CIBERSORT is a deconvolution algorithm based on gene expression data. Through a least squares regression model, it matches the transcriptome data of mixed tissue samples with the gene expression signatures of known immune cell types (LM22 signature matrix), thereby quantifying the proportions of 22 immune cell subsets (Chen et al., 2018). LM22 was downloaded from the CIBERSORT website and used to reckon the abundance of immune cells through 1,000 permutations. The LM22 data were correlated and visualized using the R software package “corrplot.” To visualize differences in immune cell populations between ovarian clear cell carcinoma and high-grade serous ovarian carcinoma control groups, the R-package “vioplot” was applied. To investigate the relationship linking biomarkers and the levels of infiltrating immune cells, Pearson correlation analysis was performed. Finally, the “ggplot2” R package was used for visualization.

### 2.10 Statistical analysis

All statistical analyses were conducted using R software (version 4.1.3). To compare the OCCC and HGSC groups, the Mann-Whitney U test was used. Furthermore, depending on the context, other methods such as LASSO regression, SVM-RFE, ROC analysis, Pearson correlation analysis, and independent samples t-tests were applied. For all statistical tests, a p-value threshold of less than 0.05 was deemed to have statistical significance.

## 3 Result

### 3.1 Expression of DIRGs in OCCC

**FIGURE 1:**
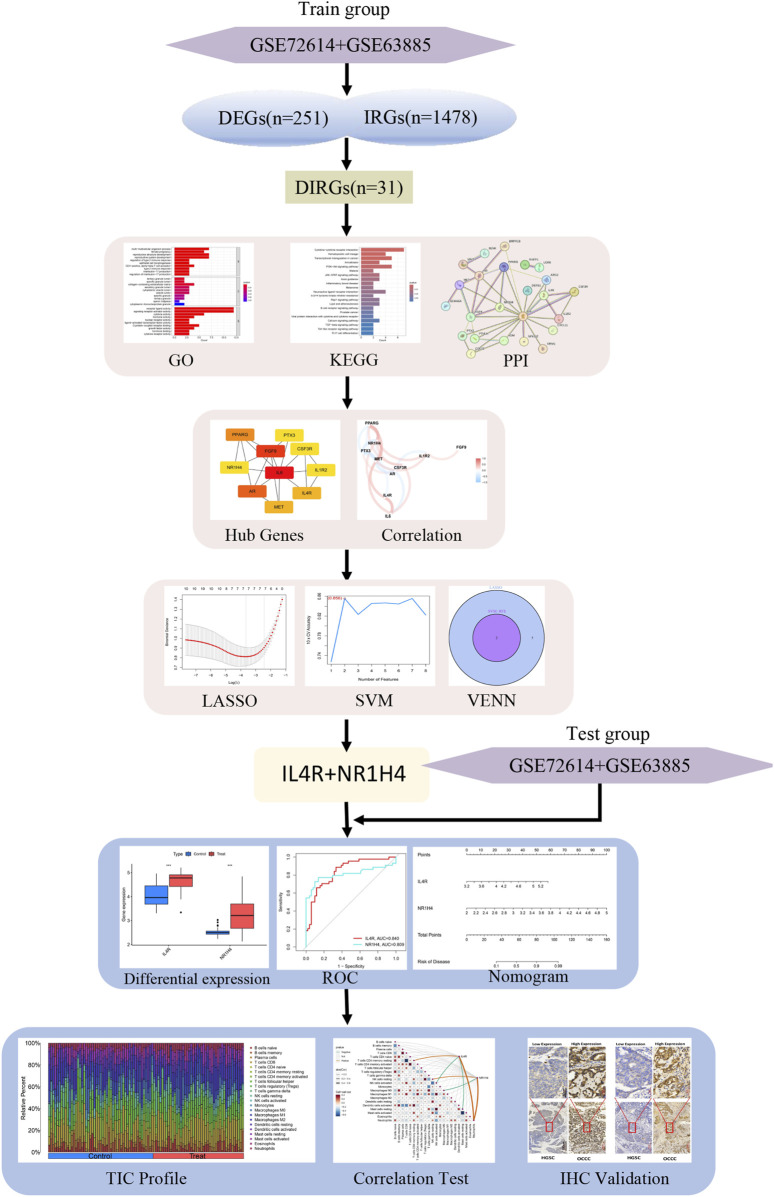
Analysis flow diagram of this study.

**FIGURE 2:**
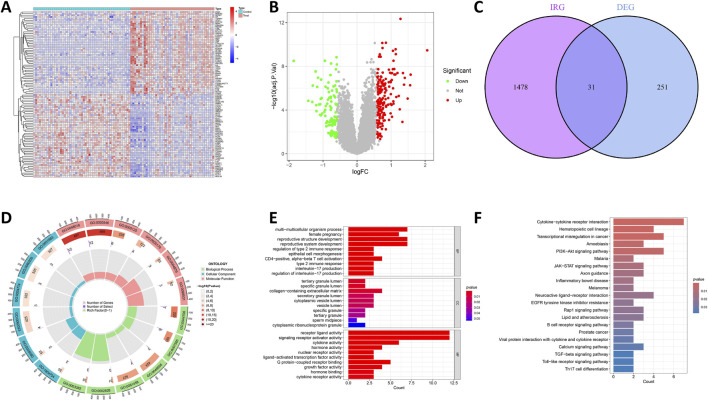
Recognition and functional enrichment of DIRGs. **(A)** A heatmap of the previous 50 DEGs identified between OCCC and HGSC from the GEO dataset, where relatively high and low gene expressions are depicted in red and blue, respectively. **(B)** A volcano plot of DEGs, with relatively high and low gene expressions represented in red and green, and gray indicates genes that are not expressed. **(C)** The intersection of DEGs and IRGs. **(D)** Circle plot of GO analysis for 31 DIRGs. **(E)** Bar graph of GO analysis for 31 DIRGs. **(F)** KEGG pathway analysis for DIRGs.

Figure 1 illustrates the workflow for analyzing DIRG expression in OCCC. By utilizing the “limma” package in R, the data sets GSE63885 and GSE73614 were analyzed, resulting in the identification of 282 DEGs using a p-value threshold of less than 0.05 (Supplementary Table S1). These DEGs were visualized as heat maps (Figure 2A). Figure 2B displays the volcano plot illustrating the distribution of differential genes between OCCC and HGSC. 180 genes were significantly upregulated, whereas 102 genes were significantly downregulated. To further investigate their functional relevance, we performed an intersection analysis between these 282 DEGs and a set of 1,509 IRGs (Supplementary Table S2), leading to the identification of 31 DIRGs (Figure 2C; Supplementary Table S3). Among them, 21 DIRGs were upregulated and 10 DIRGs were downregulated in the OCCC group.

### 3.2 GO and KEGG pathway enrichment analyses

The function of the R was analyzed using “ClusterProfiler,” and the enrichment outcomes were subsequently visualized with the “ggplot2” package to comprehensively explore the functions and pathways of the 31 DIRGs. The findings of GO analysis demonstrated that, in the context of genetic biological processes (BP), the emphasis was largely placed on multicellular organism processes, reproductive structure development, reproductive system development, and regulation of type 2 immune response. In terms of cellular components (CCs), these DIRGs were predominantly localized within the tertiary granule lumen, specific granule lumen, and collagen-containing extracellular matrix. Concerning molecular functions (MFs), they were mainly linked to receptor ligand activity, signaling receptor activator activity, cytokine activity, and hormone activity, all of which reached statistical significance at the P < 0.05 level (Figures 2D,E; Supplementary Table S4). Additionally, KEGG analysis demonstrated that DIRGs are closely associated with cytokine-cytokine receptor interactions, transcription dysregulation in cancer, and the PIK3-AKT signaling pathway (Figure 2F; Supplementary Table S5). All these findings point to a significant correlation between OCCC and immunity.

### 3.3 Building PPI network

**FIGURE 3:**
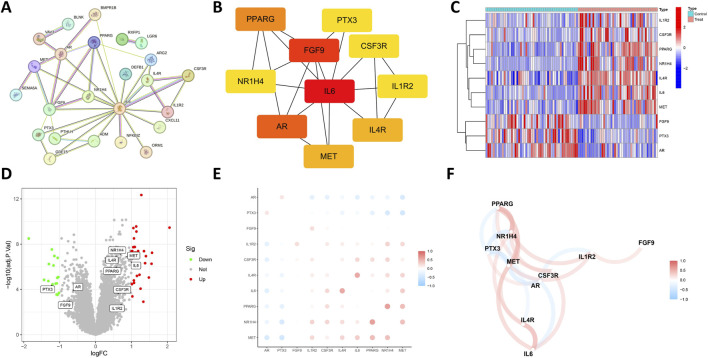
Expression correlation of the core DIRGs. **(A)** PPI network analysis of the 31 DIRGs. **(B)** Network diagram of 10 core DIRGs obtained through the MCC algorithm. **(C)** The core DIRGs expression correlation network diagram. **(C)** A heatmap of the 10 core DIRGs, where relatively high and low gene expressions are depicted in red and blue, respectively. **(D)** A volcano plot of the 10 core DIRGs, with relatively high and low gene expressions represented in red and green, and gray indicates genes that are not expressed. **(E)** Co-expression network map among the 10 core DIRGs. **(F)** A network diagram of the expression correlation of the core DIRGs. In which the darkness of the color intuitively reflects the strength of the correlation, red indicates a positive correlation, and blue indicates a negative correlation, with the darker color signifying a stronger correlation.

The STRING database was utilized to investigate the PPI among 31 DIRGs associated with OCCC. We deleted the unassociated DIRGs (Figure 3A). Subsequently, the 10 nodes associated with the MCC were obtained by clustering the network genes with the Cell Hubba plug-in in Cytoscape software. The 10 core DIRGs are FGF9, IL-6, PPARG, AR, PPARG, MET, ILAR, NR1H4, PTX3, CSF3R, and IL1R2 (Figure 3B). All the 10 core DIRGs were presented through a heatmap and volcano plot. NR1H4, IL4R, MET, PPARG, IL6, CSF3R, and IL1R2 genes exhibited high expression levels, whereas PTX3, AR, and FGF9 genes showed low expression levels in the OCCC group (Figures 3C,D).

### 3.4 Relationship of the 10 core DIRGs in OCCC

**FIGURE 4:**
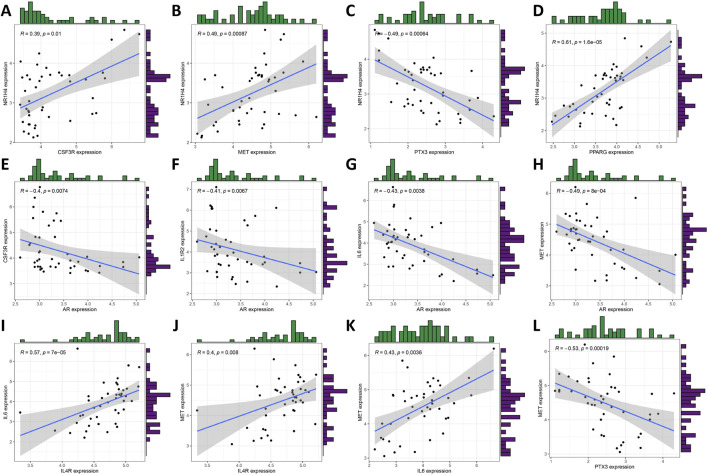
**(A–L)** Scatterplot of some highly correlated DIRGs.

Figures 3E,F illustrate the results of correlation analysis for these 10 core genes, with a filtering coefficient set at “cutoff = 0.3.” In this representation, red indicates a positive correlation, while blue represents a negative correlation. For instance, CSF3R exhibits positive correlations with NRIH4, however, AR demonstrates a negative correlation with CSF3R, IL1R2, IL6, and MET (Figures 4A–L).

### 3.5 Development and evaluation of LASSO and mSVM-RFE models

**FIGURE 5:**
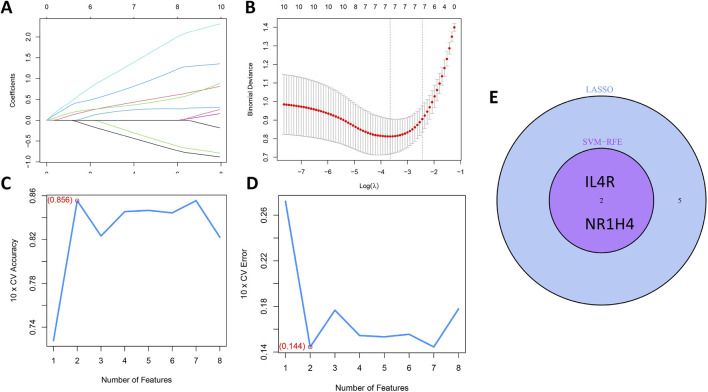
Establishment of the OCCC prediction model. **(A)** LASSO regression coefficient profiles for the 10 core DIRGs, with each curve representing the trajectory of change for an individual DIRG. **(B)** A plot of partial likelihood deviance against the logarithm (λ) using the LASSO Cox regression model. **(C)** The total within-cluster sum of squares curve under varying cluster numbers k, reaching an “elbow point” at k = 2. **(D)** The average silhouette width curve under varying cluster numbers k, with the maximum average silhouette width achieved at k = 2. **(E)** A Venn diagram displaying the overlap of candidate genes selected by the LASSO regression and the mSVM-RFE models, identifying NR1H4 and IL4R as the two key DIRGs.

The LASSO and mSVM-RFE algorithms were utilized to identify vital prognostic biomarkers in OCCC. Through the LASSO analysis, with 10-fold cross-validation for the selection of the best, a total of seven DIRGs, including PTX3, FGF9, PPARG, IL4R, IL6, MET, and NR1H4, were selected as candidate genes (Figures 5A,B). Subsequently, the mSVM-RFE model was utilized to screen these ten DIRGs, resulting in two candidate genes (IL4R and NR1H4) being identified (Figures 5C,D). By taking the intersection of the results obtained from both models (Figure 5E), we ultimately confirmed that IL4R and NR1H4 are the two most promising candidate DIRGs for OCCC.

### 3.6 The diagnostic efficacy of two critical DIRGs was assessed

**FIGURE 6:**
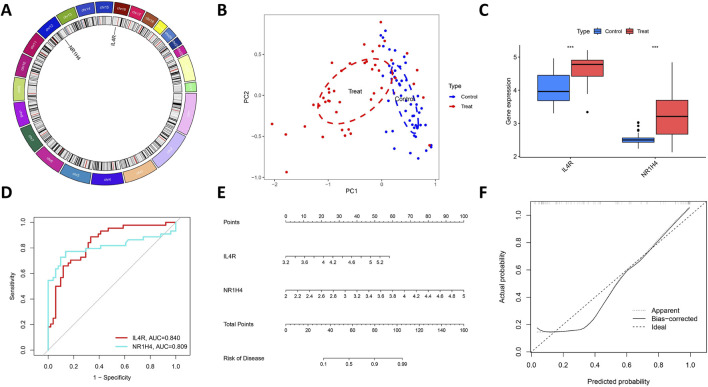
Further analysis of two key DIRGs. **(A)** The chromosomal locations of NR1H4 and IL4R. **(B)** Principal component analysis clearly distinguishes OCCC and HGSC using NR1H4 and IL4R. **(C)** The relative expression levels of NR1H4 and IL4R between OCCC and HGSC from the training group. **(D)** ROC curves validated the performance of NR1H4 and IL4R for the prediction of OCCC in the training group **(E)** Diagnostic Nomo plot of NR1H4 and IL4R. **(F)** Calibration curve of a model composed of NR1H4 and IL4R.

By examining the overlap of the Lasso regression model and the mSVM-RFE model, two genes, IL4R and NR1H4, were selected for further analysis as potential candidates. The chromosomal locations of these two genes, IL4R and NR1H4, are depicted in Figure 6A. Principal component analysis (Figure 6B) demonstrated that these two candidate genes distinctly differentiated OCCC from HGSC, suggesting their potential significance in OCCC diagnosis. Furthermore, to evaluate the predictive capability of the two candidate genes, the analysis of differential expression was carried out relying on the training datasets (GSE63885 and GSE73614). The findings revealed that patients with OCCC had markedly elevated expression levels of IL4R and NR1H4 compared to those with HGSC (Figure 6C). Additionally, the ROC curve analysis revealed AUC values of 0.840 for IL4R and 0.809 for NR1H4, both exceeding 0.7, which signifies strong diagnostic accuracy (Figure 6D). Based on these findings, we developed a nomogram integrating IL4R and NR1H4 to predict the OCCC risk (Figures 6E,F). This model provides clinicians with a reliable tool for evaluating individual patient risk, thereby enhancing early diagnosis and timely intervention.

### 3.7 The diagnostic efficacy of IL4R and NR1H4 was validated

**FIGURE 7:**
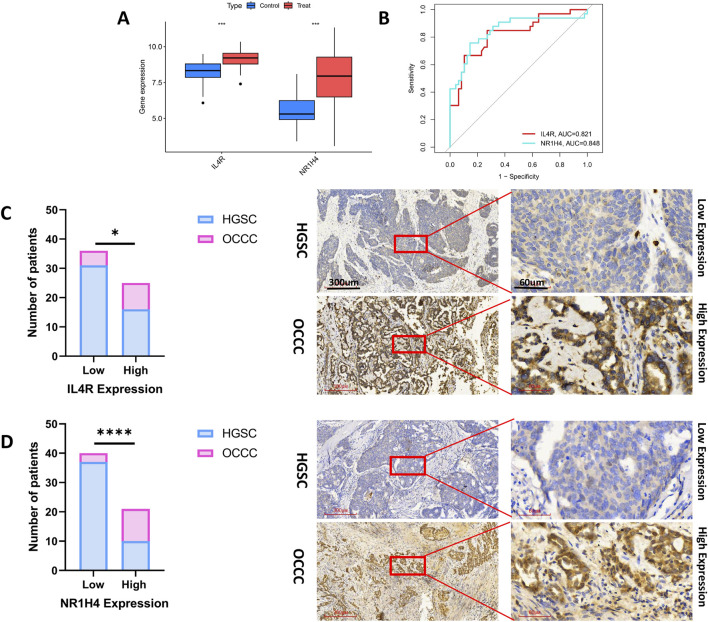
The diagnostic efficacy of IL4R and NR1H4 was validated. **(A)** In the test group (GSE65986 and GSE68600 datasets), IL4R and NR1H4 exhibit differential expression between OCCC and HGSC (treat represents the OCCC group, and control represents the HGSC group). **(B)** ROC curves for IL4R and NR1H4 in the test group. **(C)** Compared with HGSC samples, IL4R expression is significantly higher in OCCC tissues (HGSC = 47; OCCC = 14). **(D)** Compared with HGSC specimens, NR1H4 expression is significantly higher in OCCC tissues (HGSC = 47; OCCC = 14). Representative images of IL4R and NR1H4 IHC staining in OCCC and HGSC patients are displayed (×40 and ×200, high expression vs. low expression). Scale bars are indicated. *P < 0.05, ***P < 0.001, ****P < 0.0001, P-values were calculated using chi-square tests.

Figure 7A demonstrates that the expression levels of IL4R and NR1H4 in OCCC were markedly HGSC across the test datasets (GSE65986 and GSE68600). The diagnostic biomarkers IL4R and NR1H4 exhibited substantial potential for early identification of OCCC, as evidenced by AUC values of 0.821 for IL4R and 0.848 for NR1H4 (Figure 7B). IHC analysis confirmed significantly elevated expression levels of IL4R and NR1H4 in OCCC samples compared to HGSC, with P-values of 0.043 for IL4R and <0.0001 for NR1H4 (Figures 7C,D). These findings suggest that IL4R and NR1H4 possess significant diagnostic value in differentiating OCCC from HGSC.

### 3.8 IL4R and NR1H4 genes correlate with the percentage of immune cell infiltration

**FIGURE 8:**
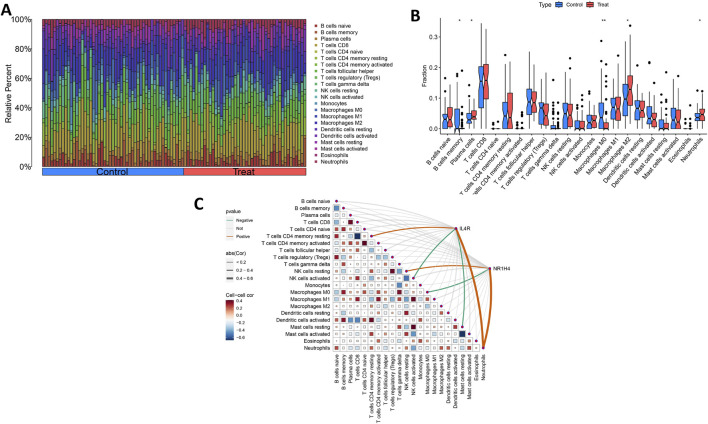
Distribution and visualization of immune cell infiltration and correlation analysis. **(A)** Bar plot showing the proportion of 22 immune cell subtypes between OCCC tissue and HGSC samples. **(B)** The violin plot shows the ratio differentiation of 22 kinds of immune cells between OCCC tissue and HGSC samples. **(C)** Correlation between IL4R and NR1H4 and infiltrating immune cells in OCCC. *P < 0.05, **P < 0.01.

Ultimately, we assessed the expression levels of the IL4R and NR1H4 genes and investigated their association with the degree of immune cell infiltration. Utilizing the CIBERSORT algorithm, we determined the relative abundances of 22 types of immune cells in both OCCC and HGSC samples (Figure 8A; Supplementary Table S6). Notably, the proportions of plasma cells, M2 macrophages, and neutrophils were elevated in the OCCC group relative to the HGSC group. Conversely, the proportion of M0 macrophages was markedly higher in the HGSC group (Figure 8B). Furthermore, IL4R exhibited positive correlations with resting T cells CD4 memory cells, and neutrophils, whereas it displayed negative correlations with activated NK cells and resting mast cells. In contrast, NR1H4 showed positive correlations with resting NK cells and neutrophils and a negative correlation with M0 macrophages (Figure 8C). IL4R and NR1H4 show a positive correlation with neutrophils. These findings concerning immune activity bolster the influence of the IL4R and NR1H4 genes.

## 4 Discussion

At present, because of the scarcity of evidence-supported therapeutic approaches for OCCC, the management protocol adheres to the National Comprehensive Cancer Network (NCCN) guidelines designed for HGSC (Armstrong et al., 2021). In contrast to HGSC, OCCC exhibits greater resistance to conventional platinum-based chemotherapy and a higher likelihood of recurrence (Ye et al., 2015a). The response rate to platinum-based therapy for OCCC ranges from 11% to 56%, compared with approximately 80% for HGSC (Ye et al., 2015b; Jiang et al., 2018; Ye et al., 2019). OCCC can be classified into two transcriptional subtypes: epithelioid and mesenchymal. The mesenchymal subtype is associated with advanced-stage disease, enriched immune-related pathways, and poor prognosis, while the epithelioid subtype is linked to early-stage tumors, frequent mutations in the Yeast mating-type switching/sucrose non-fermenting (SWI/SNF) complex, and a more favorable prognosis (Tan et al., 2019). Epithelial (EpiCC) and mesenchymal (MesCC) subtypes exhibit distinct patterns of immune infiltration. Compared to EpiCC, MesCC shows a higher level of enrichment in both immune-related pathway activity and the presence of tumor-infiltrating lymphocytes. Specifically, EpiCC expresses genes related to the regulatory T cells and the activated dendritic cells, whereas MesCC expresses genes associated with CD4 memory T cells and γδT cells. Moreover, MesCC shows expression of various immune-related genes, including those involved in antigen processing and presentation, such as CD74, HLA-DPA1, HLA-DRA, HLA-DMB, and TAP1. The prognosis of OCCC is positively associated with the expression levels of antigen-presenting genes and negatively correlated with the overexpression of immune checkpoint molecules, particularly PD-1 and CTLA-4. Gene expression profiling studies have demonstrated that effector memory CD8^+^ T cells, as well as immune checkpoint molecules such as PD-1, Tim-3, CTLA-4, and Lymphocyte-activation gene 3 (LAG3), are upregulated in OCCC tumors. Despite these insights, the immune microenvironment of OCCC remains incompletely characterized. Key immune checkpoints in OCCC include LAG-3, PD-1/PD-L1, and CTLA-4, which are expressed on various immune cells. Tumor-infiltrating lymphocytes (TILs) expressing LAG-3 compromise the effector function of PD-1+CD8^+^ T cells. However, combined inhibition of LAG-3 and PD-1 not only restores this effector function but also significantly enhances the antitumor activity (Zaitsu et al., 2023). It has been demonstrated that tumors in patients with early recurrence of OCCC exhibit increased infiltration by mast cells, neutrophils, macrophages, and T cells. This indicates that OCCC is characterized by a higher TIL signal and relatively abundant CD8^+^ T cells compared to Tregs. Furthermore, OCCC with high expression levels of CTLA-4 and PD-1 exhibits a higher recurrence rate (Huang et al., 2023). A study using multiple immunohistochemical approaches to investigate immune cells in OCCC tumors found that increased infiltration of CD8^+^ T cells and macrophages was associated with poor survival outcomes, while high PD-L1 expression correlated with improved survival (Mabuchi et al., 2010). Additionally, low expression of the ARID1A gene is linked to tumor invasion and reduced immune infiltration in OCCC. It shows a negative correlation with CD8^+^ T cells and plasma cells, yet exhibits a positive association with activated CD4^+^ T cells (Jung et al., 2021). Despite OCCC’s high resistance to radiotherapy and chemotherapy, its unique immune microenvironment makes immunotherapy, including immune checkpoint inhibitors, immunomodulatory drugs, and T cell engineering therapies, a promising new treatment modality. Further investigation into the immunological, molecular, and genetic characteristics of OCCC is expected to pave the way for more personalized treatments using specific targeted immunotherapies. For example, pembrolizumab and nivolumab plus ipilimumab induction followed by nivolumab maintenance have shown higher response rates and prolonged progression-free survival in platinum-resistant OCCC patients (Oda et al., 2018). Inhibition of Histone Deacetylase 6 (HDAC6) combined with the immune checkpoint blockade (anti-PD-L1) can treat ARID1A-mutated OCCC by enhancing cytotoxic T cell activity and modulating the tumor immune microenvironment (Fukumoto et al., 2019).

Our GO analysis results indicated that DIRGs are primarily associated with molecular functions such as receptor ligand activity, signaling receptor agonist activity, and cytokine activity. Several studies have investigated NK cells from OCCC patients who exhibit resistance to conventional chemotherapy. These studies demonstrated a cytotoxic interaction between NK cells and autologous tumor cells derived from these patients. In the presence of autologous tumor cells, the degranulation capacity of NK cells isolated from the patient’s peritoneal fluid is compromised due to inhibitory interactions between HLA-I-specific NK cell receptors and HLA-I molecules, as well as impaired interactions between activating NK receptors and their respective ligands (Patrizi et al., 2020). Additionally, KEGG pathway analysis indicated that DIRGs are associated with cytokine-cytokine receptor interaction and the PI3K-AKT signaling pathway. Research has shown that in glioma, Tripartite Motif Containing 6 (TRIM6) modulates the interaction between cytokines and their receptors, contributing to inflammatory responses and immune regulation imbalance, thereby influencing glioma progression (Guo J. et al., 2023). The PI3K/AKT/mTOR signaling cascade has been widely shown to be crucial in the tumorigenesis, chemoresistance, and radioresistance of ovarian carcinoma through various aberrations within the pathway and alterations at multiple regulatory points. Dysregulation of this pathway can contribute to tumor formation, cancer cell migration, invasion, and enhanced chemoresistance. Beyond its involvement in ovarian carcinogenesis, the PI3K/AKT/mTOR signaling pathway also protects primordial follicles from damage during oocyte maturation. The combination of the pan-PI3K inhibitor LY294002 with cisplatin effectively inhibits the proliferation and migration of ovarian cancer cells by downregulating the expression of Matrix Metalloproteinase-2 (MMP-2), TIMP1, and TIMP2 (Ediriweera et al., 2019). Overexpression of progranulin (PGRN) leads to cisplatin resistance in epithelial ovarian cancer cell lines. Studies have shown that the inhibition of the PI3K/AKT/mTOR signaling reduces PGRN expression in OCCC cells (Perez-Juarez et al., 2019). The AKT inhibitor, Perifosine, has exhibited significant antitumor activity in OCCC that has developed resistance to bevacizumab or cisplatin, thereby inhibiting proliferation and inducing apoptosis in OCCC cells (Guo D. et al., 2023). Targeted inhibition of the PI3K/AKT/mTOR signaling pathway holds promise as a therapeutic strategy for addressing OCCC recurrence and drug resistance.

Based on a machine-learning algorithm and diagnostic power analysis, we identified two diagnostic biomarkers, IL4R and NR1H4, which were validated using independent validation sets and immunohistochemical methods. IL4R, a cell surface receptor that targets M2 macrophages in the tumor microenvironment, is classified into two types: Type I and Type II. Type I IL4R is primarily found in certain immune cells and is composed of the IL4Rα and IL2Rγc subunits. IL-4 binds to the alpha subunit of IL4R, subsequently recruiting either the IL2Rγc or IL13Rα subunits (LaPorte et al., 2008). Type II IL4R comprises the IL4Rα and IL13Rα subunits and is significantly upregulated in certain tumor types associated with poor prognosis (Vadevoo et al., 2024). Elevated expression of IL4Rα has been positively correlated with the recurrence of oral squamous cell carcinoma (OSCC) patients (Kwon et al., 2015). High levels of IL4R are also linked to an increased risk of cervical cancer (Muhammad et al., 2021). Studies have demonstrated that high expression of IL4R in breast cancer bone metastasis involves monocyte-derived macrophage subsets promoting metastasis in an IL4R-dependent manner (Ma et al., 2020). Activation of the Extracellular signal-regulated kinase (ERK) pathway via IL4/IL4R signaling plays a critical role in the proliferative phase of preosteoclasts, contributing to colorectal cancer bone metastasis (Sasano et al., 2015). The IL4/IL4Rα signaling axis enhances glucose and glutamine metabolism in breast cancer cells, thereby promoting tumor growth (Jin et al., 2014). Furthermore, IL-4 cytotoxin has shown significant anti-tumor activity against IL4R-expressing ovarian carcinoma in a mouse model of ovarian cancer. The targeted therapy against IL4R has also been effective in eradicating cancer in a mouse model of orthotopic ovarian cancer (Li et al., 2014).

The farnesoid X receptor (FXR), encoded by NR1H4, belongs to the nuclear receptor superfamily and functions as a transcription factor (Ramos Pittol et al., 2020). It is essential for numerous biological processes, like bile acid metabolism, gluconeogenesis, glycogen synthesis, lipogenesis, and ammonia detoxification (Massafra and van Mil, 2018). Furthermore, FXR is proposed to be involved in the development and progression of multiple types of carcinoma, including breast carcinoma, clear cell renal carcinoma, colorectal carcinoma, and hepatocellular carcinoma. There is a notable decrease in NR1H4 expression within pathways involved in the inflammatory response, IL6/STAT3 signaling, IL2 STAT5 signaling, initial estrogen response, and Transforming Growth Factor-β(TGFβ) signaling. This downregulation may be closely linked to biliary atresia (Ma et al., 2022). Elevated NR1H4 levels correlate with poor prognosis in breast cancer patients, promoting tumor cell proliferation and metastasis through CBP-dependent p53 K382 acetylation (Huang et al., 2024). In clear cell renal cell carcinoma (CCRCC), high NR1H4 expression facilitates early diagnosis, while its knockdown significantly inhibits cancer cell proliferation, migration, and invasion. Genetic alterations and DNA methylation of NR1H4 are strongly associated with patient prognosis in clear cell renal cell carcinoma (Huang et al., 2022). In colorectal cancer, NR1H4 activation suppresses the JAK2/STAT3 signaling pathway via trans-activation of the suppressor of cytokine signaling 3 (SOCS3) gene and antagonizes Wnt/β-Catenin signaling, acting as a tumor suppressor (Yu et al., 2020). Additionally, NR1H4 enhances drug-induced cell death in colorectal cancer by modulating myelocytomatosis oncogene (MYC) expression and Myc protein stability (Huang et al., 2022). In hepatocellular carcinoma, the nuclear translocation of transketolase promotes HDAC3 binding to the NR1H4 promoter, thereby inhibiting FXR expression (Li et al., 2020).

We employed the CIBERSORT algorithm to examine the relationship between NR1H4 and IL4R gene expression and immune cell infiltration. The analysis demonstrated that the proportions of plasma cells, M2 macrophages, and neutrophils were significantly elevated in the OCCC group relative to the HGSC group, while the proportion of M0 macrophages was notably higher in the HGSC group compared to the OCCC group. Additionally, tumor tissues from patients with recurrent OCCC exhibited increased expression signals for mast cells, neutrophils, macrophages, and T cells. Notably, IL4R expression levels are significantly higher in M2 macrophages than in M1 macrophages (Movahedi et al., 2010; Shiao et al., 2015; Vadevoo et al., 2017). IL4 and IL4R can induce M2 macrophage polarization, thereby promoting tumor progression and metastasis. Targeting IL4R enhances the efficacy of nab-paclitaxel by facilitating the conversion of M2 macrophages to an M1-like phenotype via the Reactive Oxygen Species (ROS)-HMGB1-TLR4 axis. This approach increases the presence of immunostimulatory cells while reducing immunosuppressive cells within the tumor microenvironment, thus enhancing antitumor immunity and providing a novel strategy for tumor immunotherapy (Vadevoo et al., 2024). The IL-4 signaling pathway, through its receptor IL4R, may be involved in regulating the immunosuppressive microenvironment of ovarian cancer. Studies have shown that the IL-4 signaling may promote tumor immune escape by activating CD4^+^ regulatory T cells (Tregs) and inhibiting the function of NK cells (Mollaoglu et al., 2024). Single-cell sequencing studies have found that the infiltration of natural killer (NK) cells and CD8^+^ T cells in the tumor microenvironment of OCCC is associated with responses to immunotherapy, suggesting that NK cells may exert their effects through direct killing of tumor cells or regulation of other immune cells (Xia et al., 2024). After kidney transplantation, the number of CD56dimCD16+ natural killer (NK) cells co-expressing IL4R (p = 0.038) and CD56bright NK cells with this phenotype increased over time (Zhu et al., 2018). At present, no research has revealed the direct association between IL4R and NK cells or CD4^+^ T cells in OCCC. However, the infiltration levels and functional states of NK cells and CD4^+^ T cells have been confirmed to be closely related to immune escape and treatment response in OCCC. Future studies are required to integrate multi-omics data to deeply explore the potential role of IL4R in the immune microenvironment of OCCC.

NR1H4 expression also correlated with the degree of immune cell infiltration, such as CD4^+^T cells, macrophages/monocytes, and neutrophils. In CCRCC, NR1H4 functions as a mediator of tumor immunity and plays a key role in tumor metastasis and drug resistance. Furthermore, the expression of NR1H4 exhibits a significant positive correlation with the infiltration levels of CD4^+^ T cells, macrophages/monocytes, and neutrophils in CCRCC (Huang et al., 2022). FXR (NR1H4) agonist GW4064 prevents dextran sulfate sodium - parenteral nutrition (DSS-PN) - induced hepatic macrophage accumulation, hepatic expression of genes associated with macrophage recruitment and activation, and transcription of cytokines in hepatic macrophages stimulated by lipopolysaccharide *in vitro* (El Kasmi et al., 2022). After treatment with an FXR agonist, bone marrow-derived macrophages exhibit increased expression of M2 polarization-associated signaling molecules (such as those related to the retinoic acid receptor pathway), while inhibition of the retinoic acid receptor pathway blocks FXR-mediated M2 polarization (Jaroonwitchawan et al., 2023). In the intestine, FXR inhibits inflammation-associated tumor growth by regulating macrophage recruitment, polarization, and interactions with Th17 cells. Deletion of FXR in intestinal macrophages exacerbates inflammation, whereas activation of FXR reduces pro-inflammatory cytokine secretion and improves intestinal barrier function (Dong et al., 2024). Additionally, FXR may indirectly influence macrophage phenotypes by regulating bile acid metabolism, such as abnormal bile acids that can trigger pro-inflammatory responses through macrophage-intrinsic FXR (Dong et al., 2024). While no study has directly reported the regulation of resting natural killer (NK) cells by FXR, evidence suggests a potential link between the two. CXCR6+ macrophages enhance interactions between T/NK cells and myeloid cells through the CXCL16-CXCR6 axis (Lu et al., 2024). FXR primarily participates in immune regulation by regulating macrophage polarization, while its effects on NK cells may be mediated indirectly through macrophages. The direct association between resting NK cells and FXR has not been explicitly supported by existing literature and requires further research for validation.

Nevertheless, our study has certain limitations. Owing to the rarity of clear cell carcinoma and the limited availability of clinical cases, the roles of NR1H4 and IL4R in OCCC and their associations with immune cell infiltration require further validation through prospective studies with larger sample sizes. Additionally, because of the current sample size constraints, only IHC was employed for verification. Future research should consider combining single-cell RNA sequencing and multi-color flow cytometry in follow-up studies to deeply dissect the effects of the IL4R and NR1HR on the functions of immune cell subsets and animal models.

## 5 Conclusion

In summary, this research utilized machine learning approaches to explore the possible relationship between immune-related genes and OCCC, uncovering a substantial connection. The immune system is crucial for both the development and progression of OCCC. Specifically, NR1H4 and IL4R were identified as key immune genes associated with macrophage M2 polarization. These findings offer novel perspectives for predicting, diagnosing, and elucidating the pathogenesis of OCCC. The importance of specific immune cell subsets within the tumor microenvironment was further highlighted. These results not only afford new insights into the immunological characteristics of OCCC but also have important implications for developing innovative immunotherapy strategies against OCCC. By identifying and validating immune genes closely linked to OCCC development, we have provided valuable information for future research and clinical practice, thereby facilitating the advancement of personalized treatment approaches.

## Data Availability

The datasets presented in this study can be found in online repositories. The names of the repository/repositories and accession number(s) can be found in the article/Supplementary Material.
